# Supplementary material for: Spikebench: An open benchmark for spike train time-series classification
Source: PLoS Comput Biol. 2023 Jan 10;19(1):e1010792. doi: 10.1371/journal.pcbi.1010792 (PMC9870156; doi:10.1371/journal.pcbi.1010792)
Supplement: S1 Text — Section A: hyperparameter values used for different classification models in this work; Section B: The list of most discriminative time series features as obtained by the feature importance analysis on spikebench datasets. Table A: Geometric mean score obtained for the XceptionTime architecture trained on the retinal stimulus classification dataset with alterations in training hyperparameters. Fig A: Metric value evolution during training of an XceptionTime model on the retina dataset with different data preprocessing strategies: blue—no preprocessing, original ISI sequences are used as input; red—standard scaling is performed before feeding the time series to the CNN; green—log-transform (f(x) = log(x + 1)) and standard scaling is applied to the input time series. Top left—training set loss evolution, top right—testing set loss evolution, bottom left—Cohen’s kappa score evolution on the test set, bottom right—test set AUC-ROC evolution during training. One can observe diverging test set loss in cases of no preprocessing or just standard scaling, at the same time training metrics are well-behaved when the log transform is applied to the data. Fig B: Boxplots of tsfresh-extracted feature distributions for features with high discriminative power as detected by the trained decision tree ensemble classifiers in the retinal stimulus type prediction task. A two-sided Mann-Whitney-Wilcoxon test with Bonferroni correction is performed to assess statistical significance; **** denotes p < 1e-4. (PDF) [file pcbi.1010792.s001.pdf]

A. Classifier hyperparameter values.

Listed below are hyperparameter values and implementation references for all of the classifier types we used. For more reference, see the example script.

- Random Forest: sklearn implementation, $n\_estimators = 500$, $max\_depth = 10$.
- Extra Trees Classifier: sklearn implementation, $n\_estimators = 500$, $max\_depth = None$ (no limit on depth).
- Logistic Regression: sklearn implementation, l_2 penalty, $C = 0.001$
- XGBoost: xgboost implementation
  - $max\_depth = 8$
  - $learning\_rate = 0.1$
  - $n\_estimators = 500$
  - $objective = binary : logistic$
  - $booster = gbtree$
  - $gamma = 0$
  - $min\_child\_weight = 1$
  - $max\_delta\_step = 0$
  - $subsample = 0.7$
  - $colsample\_bytree = 1$
  - $colsample\_bylevel = 1$
  - $colsample\_bynode = 1$
  - $reg\_alpha = 0$
  - $reg\_lambda = 1$
  - $scale\_pos\_weight = 1$
  - $base\_score = 0.5$
- FCN, InceptionTime, XceptionTime, ResNet: tsai implementation
  - $epochs = 200$
  - $max\_lr = 0.1$
  - $optimizer = sgd$
  - $weight\_decay = 1e - 4$
  - $batch\_size = 128$

- *lr_schedule* = *cosine*
- *best_model* = with the largest *cohen_kappa* or *accuracy* on the validation set

We have looked at how the performance of a CNN is robust to the above training hyperparameters using the XceptionTime architecture on the retinal stimulus classification dataset (see Table A in S1 Text).

Varied hyperparameters w.r.t. default setting	Geometric mean score
Default setting	0.9270
Peak lr = 0.01	0.8978
Adam optimizer, peak lr = 0.01	0.9048
One cycle lr schedule	0.9095
100 training epochs	0.9204
Batch size = 256	0.9198

Table A. Geometric mean score obtained for the XceptionTime architecture trained on the retinal stimulus classification dataset with alterations in training hyperparameters.

We found that generally the CNN performance is not significantly affected by the changes in the main hyperparameters, being relatively robust with respect to these changes. We do, however, expect that a thorough hyperparameter search (including search over the architecture's parameters) could result in a significant improvement in performance.

B. Discriminative tsfresh features.

In order to select the important groups of discriminative features, we trained several random forest classification models (with different random seeds) on each dataset and used the feature importance scores extracted from the classifiers to rank the *tsfresh* features. We have used the mean scores averaged over different datasets to identify the features that are discriminative for spiking data across different scenarios. According to this feature ranking procedure, the following groups of *tsfresh* features are selected (see also Fig B in S1 Text):

- *median*, *kurtosis*, *quantile_q* – simple statistics of the ISI value distribution in the series like the median ISI value, q quantiles and kurtosis of the ISI value distribution

- *change_quantiles* – this feature is calculated by fixing a corridor of the time series values (defined by lower and higher quantile bounds, q_l and q_h, which are hyperparameters), then calculating a set of consecutive change values in the series (differencing) and then applying an aggregation function (mean or variance). Another boolean hyperparameter *is_abs* determines whether absolute change values should be taken or not.

- $fft\_coefficient$ – absolute values of the fast Fourier transform coefficients (individual coefficient values and aggregates).

- *entropy* – values of the sample entropy, the approximate entropy and the binned entropy of the power spectral density of the time series.

- *agg_linear_trend* – features from linear least-squares regression (standard error in particular) for the values of the time series that were aggregated over chunks of a certain size (with different aggregation functions like min, max, mean and variance). Chunk sizes vary from 5 to 50 points in the series.

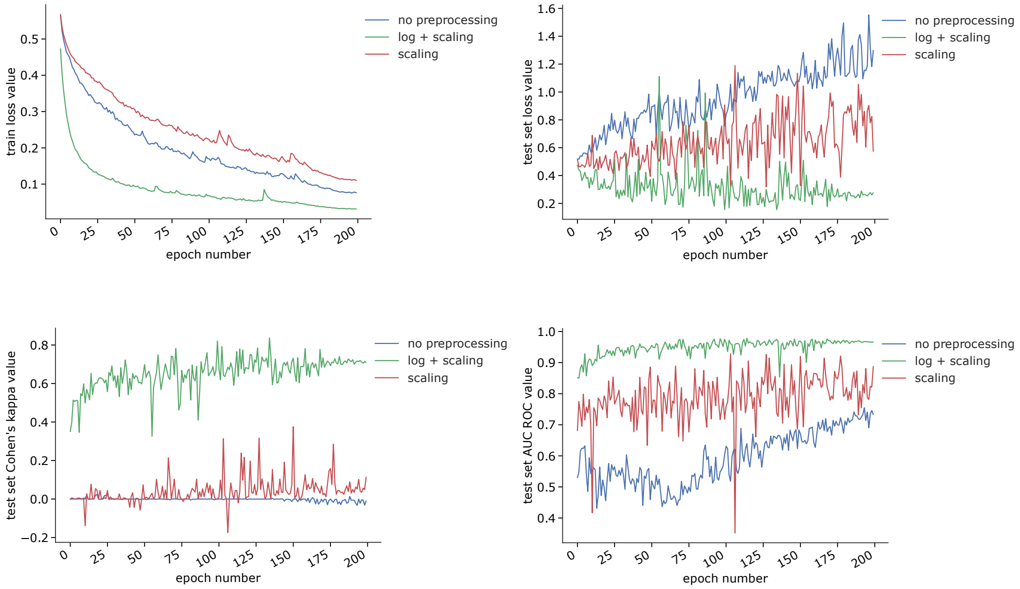

Fig A. Metric value evolution during training of an XceptionTime model on the retina dataset with different data preprocessing strategies: blue – no preprocessing, original ISI sequences are used as input; red – standard scaling is performed before feeding the time series to the CNN; green – log-transform ($f(x) = \log(x + 1)$) and standard scaling is applied to the input time series. Top left – training set loss evolution, top right – testing set loss evolution, bottom left – Cohen's kappa score evolution on the test set, bottom right - test set AUC-ROC evolution during training. One can observe diverging test set loss in cases of no preprocessing or just standard scaling, at the same time training metrics are well-behaved when the log transform is applied to the data.

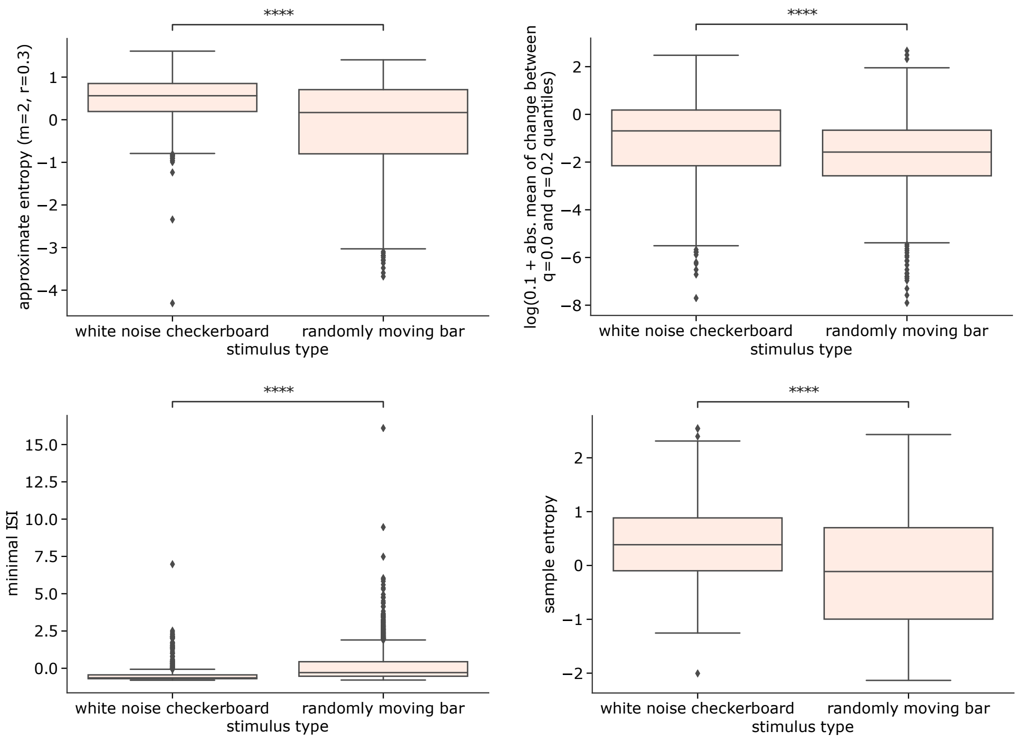

Fig B. Boxplots of *tsfresh*-extracted feature distributions for features with high discriminative power as detected by the trained decision tree ensemble classifiers in the retinal stimulus type prediction task. Two-sided Mann-Whitney-Wilcoxon test with Bonferroni correction is performed to assess statistical significance; **** denotes $p < 1e-4$.
